# Temperature-dependent structural plasticity of hippocampal synapses

**DOI:** 10.3389/fncel.2022.1009970

**Published:** 2022-10-19

**Authors:** Zhendong Feng, Lopamudra Saha, Clio Dritsa, Qi Wan, Oleg O. Glebov

**Affiliations:** ^1^Department of Pathophysiology, Institute of Neuroregeneration and Neurorehabilitation, School of Basic Medicine, Qingdao University, Qingdao, China; ^2^Wolfson Centre for Age-Related Diseases, The Institute of Psychiatry, Psychology and Neuroscience, King’s College London, London, United Kingdom; ^3^Department of Old Age Psychiatry, The Institute of Psychiatry, Psychology and Neuroscience, King’s College London, London, United Kingdom

**Keywords:** actin cytoskeleton, GABA, hypothermia, synapse, synaptic plasticity, neurodegeneration, hyperthermia

## Abstract

The function of the central nervous system (CNS) is strongly affected by temperature. However, the underlying processes remain poorly understood. Here, we show that hypothermia and hyperthermia trigger bidirectional re-organization of presynaptic architecture in hippocampal neurons, resulting in synaptic strengthening, and weakening, respectively. Furthermore, hypothermia remodels inhibitory postsynaptic scaffold into enlarged, sparse synapses enriched in GABAA receptors. This process does not require protein translation, and instead is regulated by actin dynamics. Induction of hypothermia *in vivo* enhances inhibitory synapses in the hippocampus, but not in the cortex. This is confirmed by the proteomic analysis of cortical synapses, which reveals few temperature-dependent changes in synaptic content. Our results reveal a region-specific form of environmental synaptic plasticity with a mechanism distinct from the classic temperature shock response, which may underlie functional response of CNS to temperature.

## Introduction

Temperature controls all biochemical and biophysical processes in the living cell. On the molecular level, rapid responses to changes in temperature occur due to the fundamental temperature dependence of chemical reactions, as defined by Lower (2013). The sum of these molecular changes underlies temperature response on the cellular level, whereby complex processes such as gene expression, cytoskeleton dynamics, and metabolism are affected on the longer timescale, resulting in manifold and wide-ranging changes across the cell. Besides the classical temperature shock response (Lindquist, 1986; Fujita, 1999), the mechanisms and functional relevance of these processes remain poorly understood.

Temperature can have profound effects on the central nervous system (CNS) (Bracho and Orkand, 1972; Weight and Erulkar, 1976; Pyott and Rosenmund, 2002; Volgushev et al., 2004; Micheva and Smith, 2005; Young et al., 2006; Kim and Connors, 2012), especially on the neuronal synapse. At the level of the synapse, temperature rapidly affects neurotransmitter release (Weight and Erulkar, 1976; Pyott and Rosenmund, 2002), synaptic vesicle (SV) cycling (Micheva and Smith, 2005; Kushmerick et al., 2006), neuronal excitability (Young et al., 2006; de la Peña et al., 2012; Hook, 2020), receptor kinetics (Mokrushin et al., 2014; Gotoh et al., 2020), and short-term synaptic plasticity (Klyachko and Stevens, 2006). In the longer term, low temperature (hypothermia) is generally associated with synaptic disassembly (Popov and Bocharova, 1992; Magariños et al., 2006; von der Ohe et al., 2006, 2007; Peretti et al., 2015). Such profound structural remodeling of the synapse is likely to exert long-term effects on synaptic functionality, with implications for CNS function. The details of temperature-induced synaptic remodeling, however, remain largely unknown.

Accumulating evidence from disease models and the clinic has established translational relevance of temperature-dependent synaptic plasticity in the context of major neurological disorders. One key area is hypothermia-associated neuroprotection in ischemic disorders including stroke, cardiac arrest, and traumatic brain injury (TBI) (Marion et al., 1997; Hemmen and Lyden, 2009; Cooper et al., 2018; Huber et al., 2019; Baker et al., 2021). Furthermore, cold shock response has been associated with protection of synapses in animal models of Alzheimer’s and prion disease (Peretti et al., 2015), implicating temperature-dependent synaptic remodeling in neurodegenerative disorders. Hyperthermia, on the other hand, has been correlated with adverse outcomes in several neurological disorders including stroke, febrile seizures and epilepsy, and TBI (Lundgren et al., 1994; Thompson et al., 2003; Kiyatkin, 2004; Walter and Carraretto, 2016). Notably, even short-term hyperthermia may induce temporary neurological damage and have long-term effects on some forms of synaptic plasticity (Bouges et al., 1987; Chen et al., 1999; Walter and Carraretto, 2016; Erkanli Senturk et al., 2020), underscoring the translational value of understanding temperature-dependent synaptic plasticity.

The cell biological mechanisms underlying temperature synaptic plasticity remain poorly understood. Most of the interest so far has centered on the potential role of canonical heat and cold shock pathways, given evidence for temperature-induced CNS expression of certain cold and heat shock proteins (Bechtold et al., 2000; Chen and Brown, 2007; Peretti et al., 2015; Sertel et al., 2021). However, the involvement of these pathways in regulation of synaptic structure and function remains unclear. An alternative candidate mechanism may be cytoskeletal dynamics, given that cytoskeletal dynamics are rapid and temperature-dependent (Fan et al., 2015), and play key regulatory roles in structural synaptic plasticity (Hotulainen and Hoogenraad, 2010; Dent, 2017); furthermore, some cytoskeletal proteins undergo regulation during hibernation (Hindle and Martin, 2013). The role of cytoskeletal dynamics in regulation of temperature-dependent synaptic remodeling, however, has not been experimentally assessed.

Here, we performed a systemic study of synaptic remodeling induced by hypothermia and hyperthermia. Our results reveal manifold effects of temperature on the structure and function of the synapse. We also describe a novel form of plasticity for hippocampal inhibitory synapses that does not require translation, but instead relies on cytoskeletal dynamics.

## Results

### Hypothermia rapidly induces presynaptic and postsynaptic enhancement in hippocampal neurons

To recapitulate the key aspects of hypothermia-induced synaptic remodelling in vitro, we employed immunostaining (Supplementary Table 1) in dissociated rat hippocampal culture, which forms a relatively homogenous neuronal population enriched with pyramidal cells and represents a well-defined model of synaptic plasticity. Previous studies have utilized various experimental systems and employed widely disparate cooling regimes and time scales both *in vitro* and *in vivo*, ranging from 4 to 35°C and minutes to days, respectively (Roelandse and Matus, 2004; Micheva and Smith, 2005; Kushmerick et al., 2006; Watanabe et al., 2014). We opted for the 3 h protocol for cooling to room temperature (18–22°C as observed throughout the study), as this was broadly consistent with more recent studies of structural plasticity in rodent neurons (Watanabe et al., 2014; Peretti et al., 2015) and did not compromise the viability of our cultures (Supplementary Figure 1A) or significantly affect the pH levels of the buffered medium (data not shown).

**FIGURE 1:**
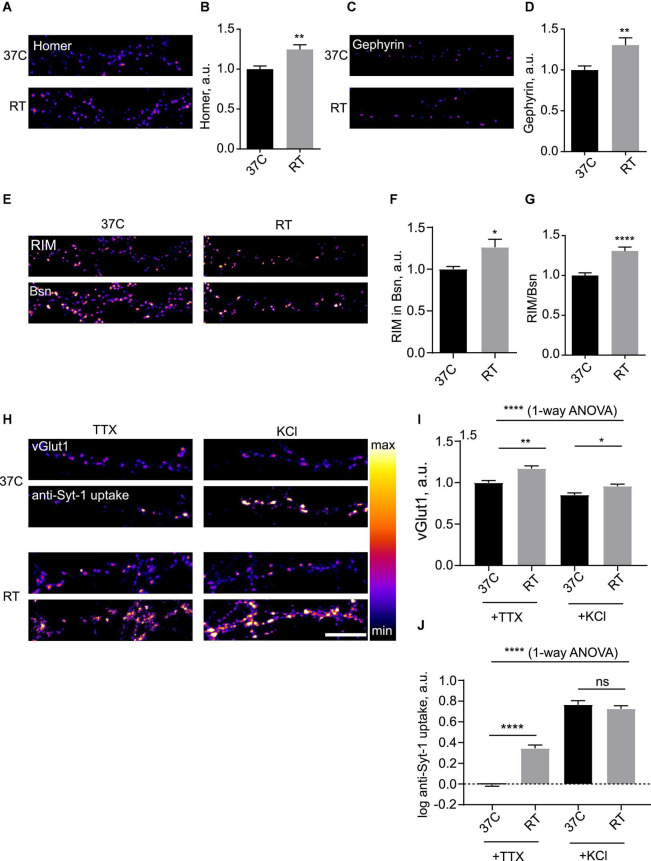
Hypothermia rapidly induces presynaptic and postsynaptic enhancement in hippocampal neurons. **(A)** Hippocampal neurons (DIV 15-21) were kept at 37°C or subjected to room temperature (RT, 18–22°C) for 3 h, fixed and stained for excitatory synapse marker Homer. **(B)** Quantification of the effect of RT treatment on synaptic levels of Homer. *N* = 15 fields of view, three independent experiments. **P < 0.01, Mann–Whitney test. **(C)** Hippocampal neurons were kept at 37°C or subjected to RT for 3 h, fixed and stained for inhibitory synapse marker Gephyrin. **(D)** Quantification of the effect of RT treatment on synaptic levels of Gephyrin. *N* = 15 fields of view, 3 independent experiments. **P < 0.01, Mann–Whitney test. **(E)** Cells were maintained at 37°C or subjected to RT for 3 h, fixed and stained for presynaptic active zone markers RIM and Bassoon (Bsn). **(F)** Quantification of the effect of RT treatment on synaptic levels of RIM. *P < 0.05, Mann-Whitney test. **(G)** Quantification of the effect of RT treatment on synaptic enrichment of RIM relative to Bsn. *N* = 15 fields of view, three independent experiments. *P < 0.05, ****P < 0.0001, Mann–Whitney test. **(H)** Hippocampal neurons were kept at 37°C or subjected to RT for 3 h, then incubated with anti-Synaptotagmin 1 antibody for 15 min either in 2 uM TTX or 50 mM KCl, fixed and stained for synaptic vesicle (SV) marker vGlut1. **(I)** Quantification of the effect of RT treatment on synaptic levels of vGlut1. ****P < 0.0001, 1-way ANOVA; **P < 0.01, *P < 0.05, Dunn’s multiple comparisons test. **(J)** Quantification of the effect of RT treatment on uptake of anti-Syt1. *N* = 15 fields of view, three independent experiments. ****P < 0.0001, 1-way ANOVA; ****P < 0.0001, ns not significant, Dunn’s multiple comparisons test. Scale bar, 10 μm.

Hypothermia led to multiple changes in synaptic organization, as evidenced by changes in levels of synaptic proteins within the synaptic puncta (Figure 1 and Supplementary Figure 1). A significant increase in the synaptic levels of the excitatory postsynaptic density (PSD) scaffolding protein Homer (Figures 1A,B) and the inhibitory PSD protein Gephyrin (Figures 1C,D) was observed. Considering the active zone (AZ), levels of large AZ matrix proteins Bassoon (Bsn) and Piccolo were increased (Supplementary Figures 1B,C). Synaptic accumulation of a key AZ adapter protein RIM was also significantly enhanced, both in absolute terms and in terms of enrichment over Bsn (Figures 1E–G). Levels of an excitatory SV marker vesicular glutamate transporter vGlut1 were increased as well (Figures 1H,I), as were the levels of an inhibitory SV marker vGAT (Supplementary Figure 1D), suggesting that presynaptic enhancement was likely present in both excitatory and inhibitory synapses. Conversely, synaptic levels of another key AZ protein, a presynaptic voltage gated calcium channel Cav2.1 (P/Q-type), were unchanged (Supplementary Figure 1E).

To assess the impact of hypothermia on synaptic function, we live-labeled neurons with an anti-Synaptotagmin1 antibody (anti-Syt1) to measure SV cycling (Matteoli et al., 1992); this assay allows to quantify anti-Syt1 uptake in presence of tetrodotoxin (TTX) as spontaneous SV cycling, whereas anti-Syt1 uptake in presence of 50 mM KCL reveals activity-dependent SV cycling (Sara et al., 2005), thus providing a readout of synaptic function. To label the total SV pool, we used immunocytochemistry for SV marker glutamate transporter vGlut1. Anti-Syt-1 uptake into Glut-1-positive puncta in presence of TTX was several-fold stronger in cooled cells, indicative of enhanced spontaneous cycling of SV, while activity-dependent anti-Syt1 uptake was unchanged (Figures 1H,J). Measurement of surface synaptic levels of Syt-1 showed a modest increase of Syt-1 staining after hypothermia, likely representing residual SV cycling at room temperature (RT) (Supplementary Figure 1F). It can therefore be concluded that mild hypothermia results in increase in the total SV pool and enhancement of presynaptic function.

### Hyperthermia induces presynaptic attenuation

**FIGURE 2:**
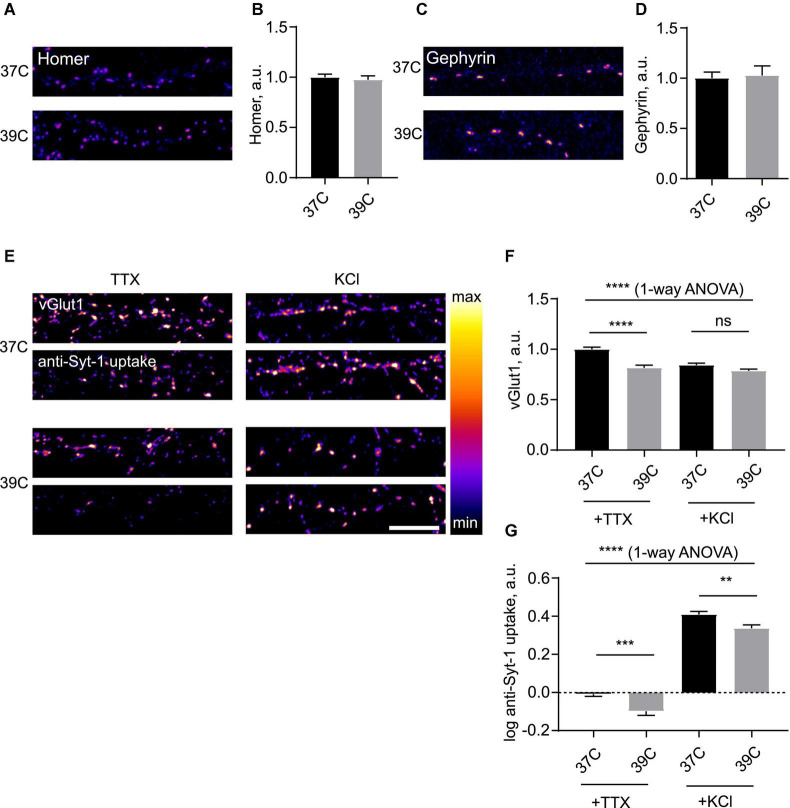
Mild hyperthermia selectively induces presynaptic disassembly. **(A)** Hippocampal neurons were kept at 37°C or 39°C for 2 h, fixed and stained for Homer. **(B)** Quantification of the effect of hyperthermia on synaptic levels of Homer. *N* = 60 fields of view, six independent experiments. ns–not significant, Mann–Whitney test. **(C)** Cells were kept at 37°C or 39°C for 2 h, fixed and stained for Gephyrin. **(D)** Quantification of the effect of hyperthermia on synaptic levels of Gephyrin. *N* = 15 fields of view, three independent experiments. ns–not significant, Mann–Whitney test. **(E)** Cells were kept at 37 or 39°C overnight, then incubated with anti-Synaptotagmin 1 antibody for 15 min either in 2 uM TTX or 50 mM KCl, fixed and stained for vGlut1. **(F)** Quantification of the effect of room temperature (RT) treatment on synaptic levels of vGlut1. ****P < 0.0001, 1-way ANOVA; ****P < 0.0001, ns - not significant, Dunn’s multiple comparisons test. **(G)** Quantification of the effect of RT treatment on uptake of anti-Syt1. *N* = 50 fields of view, five independent experiments. ****P < 0.0001, 1-way ANOVA; ***P < 0.001, **P < 0.01, Dunn’s multiple comparisons test. Scale bar, 10 μm.

We then decided to investigate the dynamics of synapses during hyperthermia. To this end, we subjected neuronal cells to various increased temperatures. Incubation of cultured neurons at temperatures equal to and exceeding 40°C led to rapid cell death (data not shown), precluding investigation of synaptic effects. We therefore opted for a 39°C temperature regime, which corresponds to mild fever (Ogoina, 2011). Overnight incubation at 39°C had no significant effect on cell viability (Supplementary Figure 1G). There were no effects of 39°C on synaptic levels of Homer or Gephyrin even after the overnight treatment with 39°C (Figures 2A–D), indicating that postsynaptic structure of excitatory and inhibitory synapses remains stable during hyperthermia. Conversely, vGlut1 was decreased, suggesting that mild fever may result in alteration in presynaptic structure and function (Figures 2E,F); this effect was noticeable after as little as 2 h at 39°C (data not shown).

To assess the effect of mild hyperthermia on synaptic function, we again employed the anti-Syt-1 uptake assay. Following ON incubation at 39°C, both spontaneous and activity-dependent anti-Syt-1 uptake were significantly decreased following hyperthermic treatment, indicative of weakened SV cycling (Figure 2G). It can therefore be concluded that mild hyperthermia results in attenuation of presynaptic function.

### A translation-independent mechanism for hypothermia-induced postsynaptic inhibitory enhancement

**FIGURE 3:**
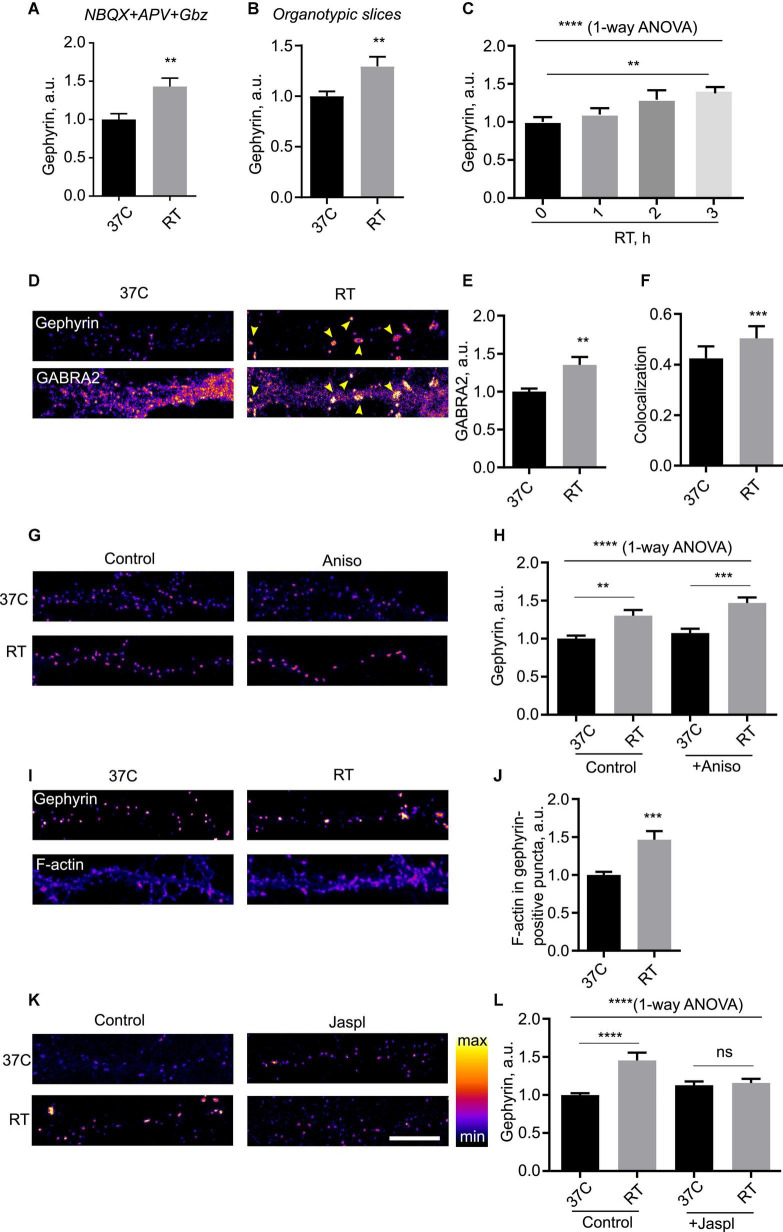
Mechanism of hypothermia-induced remodeling of inhibitory synapses. **(A)** Cells were kept at 37°C or room temperature (RT) for 3 h in presence of a silencing mix of 100 μM NBQX, 50 μM APV and 50 μM Gabazine, fixed and immunostained for Gephyrin. *N* = 15 fields of view from three independent experiments. **P < 0.01, Student’s *t*-test. **(B)** Rat organotypic slices were incubated at 37°C or RT for 3 h, fixed and immunostained for Gephyrin. **P < 0.01, Student’s *t*-test. *N* = 14 fields of view, two independent experiments. **(C)** Cells were incubated at RT for indicated amounts of time, fied, and immunostained for Gephyrin. *N* = 15 fields of view from three independent experiments. ****P < 0.0001, 1-way ANOVA; **P < 0.01, Dunn’s multiple comparisons test. **(D)** Cells were maintained at 37°C or subjected to RT for 3 h, fixed and immunostained for Gephyrin and GABA receptor alpha subunit (GABRA2). Arrowheads denote increased accumulation of GABRA2 into Gephyrin-positive puncta. **(E)** Quantification of the effect of RT treatment on synaptic recruitment of GABRA2. **P < 0.01, Student *t* test. **(F)** Colocalization between Gephyrin and GABRA2 in 37°C vs. RT-treated cells. ***P < 0.001, Student’s *t*-test. **(G)** Cells were kept at 37°C or subjected to RT for 3 h in presence of translation blocker anisomycin (100 μg/ml), fixed and immunostained for Gephyrin. **(H)** Quantification of the effect of anisomycin; *N* = 15 fields of view from three independent experiments. ****P < 0.0001, 1-way ANOVA; **P < 0.01, ***P < 0.001, Dunnett’s post-test. **(I)** Cells were kept at 37°C or subjected to RT for 3 h, then stained with AF-647-conjugated phalloidin to label filamentous (F)-actin and gephyrin. **(J)** Quantification of the effect of hypothermia on F-actin levels in gephyrin-positive puncta. ***P < 0.001, Student *t*-test. **(K)** Cells were kept at 37°C or subjected to RT for 3 h in presence of F-actin stabilizer jasplakinolide (jaspl, 1 μM) for 3 h, fixed and immunostained for Gephyrin. **(L)** Quantification of the effect of jasplakinolide. *N* = 15 fields of view from three independent experiments. ****P < 0.0001, 1-way ANOVA; ****P < 0.0001, ns - not significant, Dunn’s multiple comparisons test. Scale bar, 10 μm.

Next, we decided to investigate hypothermia-induced plasticity of inhibitory synapses in more detail. Blockade of synaptic transmission using a cocktail of receptor inhibitors did not alter the temperature effect (Figure 3A), indicating that neurotransmission was not required for cooling-induced plasticity. Cooling-induced increase in synaptic Geph was also evident in organotypic rat brain slices, suggesting that the observed phenomenon was unlikely to be an artifact of dissociated culture preparation (Figure 3B). Time-course study of the effect showed that it gradually developed over several hours, implying a timescale consistent with other forms of homeostatic plasticity such as synaptic scaling (Ibata et al., 2008; Turrigiano, 2008; Figure 3C). The effect was accompanied by the increase in the synaptic area indicative of a structural rearrangement within the inhibitory synapse (Supplementary Figure 2A,B). The nearest neighbor distance for inhibitory synapses, on the other hand, was increased, indicative of a sparser spatial distribution for enlarged synapses (Supplementary Figure 2C).

To gain insight into the functional relevance of hypothermia-induced enhancement of inhibitory synapses, we performed dual immunocytochemistry for Geph and a major GABAA receptor subunit 2 (GABRA2). The enhancement in the synaptic Geph levels was closely matched by the increased synaptic levels of GABRA2, which colocalizes with Geph puncta in hippocampus (Figures 3D,E and Supplementary Figure 3A); colocalization between GABRA2 and Geph was significantly increased by cooling, suggesting that the enlarged Geph-positive puncta specifically recruited GABA receptors to the synapse (Figure 3F). Increased recruitment of GABA receptors to the inhibitory synapses suggested functional relevance of cooling-induced enhancement of PSDs.

We next set out to identify the mechanisms responsible for cooling-induced synaptic remodeling. Live labeling with a fluorescent plasma membrane presynaptic marker Cholera Toxin B subunit showed no obvious difference in membrane organization, suggesting that the effects of cooling were likely not associated with changes in plasma membrane structure (Supplementary Figure 3B). To test whether the synaptic remodeling of inhibitory proteins required protein translation as per canonical temperature shock response (Lindquist, 1986; Fujita, 1999), we assessed the effect of cooling on Geph in the presence of translation elongation blocker Anisomycin (Aniso). Strikingly, this treatment did not affect the effect of cooling (Figures 3G,H), suggesting that the temperature-dependent plasticity of inhibitory synapses did not require translation.

An alternative candidate mechanism may involve reorganization of actin cytoskeleton, which regulates inhibitory synapse dynamics (Hanus et al., 2006) and temperature dependence *in vitro* (Pollard, 1976); of note, actin dynamics have been implicated in regulation of dendritic spines in hypothermia (Gisselsson et al., 2005). We therefore tested the effect of cooling on the actin dynamics in neurons. Cooling resulted in a significant increase in accumulation of polymerized filamentous (F)-actin in the inhibitory synapse as shown by overlap with Geph (Figures 3I,J); increased accumulation of F-actin was also observed in the cell soma (data not shown). To assess the importance of actin dynamics for temperature-induced synaptic remodeling, we pharmacologically blocked actin turnover by adding F-actin stabilizing drug jasplakinolide. Previous reports indicated that pharmacological manipulation of actin dynamics by Jasplakinolide is sufficient to affect the structure of inhibitory synapses (Kirsch and Betz, 1995); indeed, we observed a small increase in Geph intensity at 37°C which, however, did not reach statistical significance. Jaspl application abolished the effect of cooling (Figures 3K,L), indicating that actin dynamics were required for cooling-induced reorganization of inhibitory synapses.

### Hypothermia-induced remodeling of hippocampal synapses *in vivo*

**FIGURE 4:**
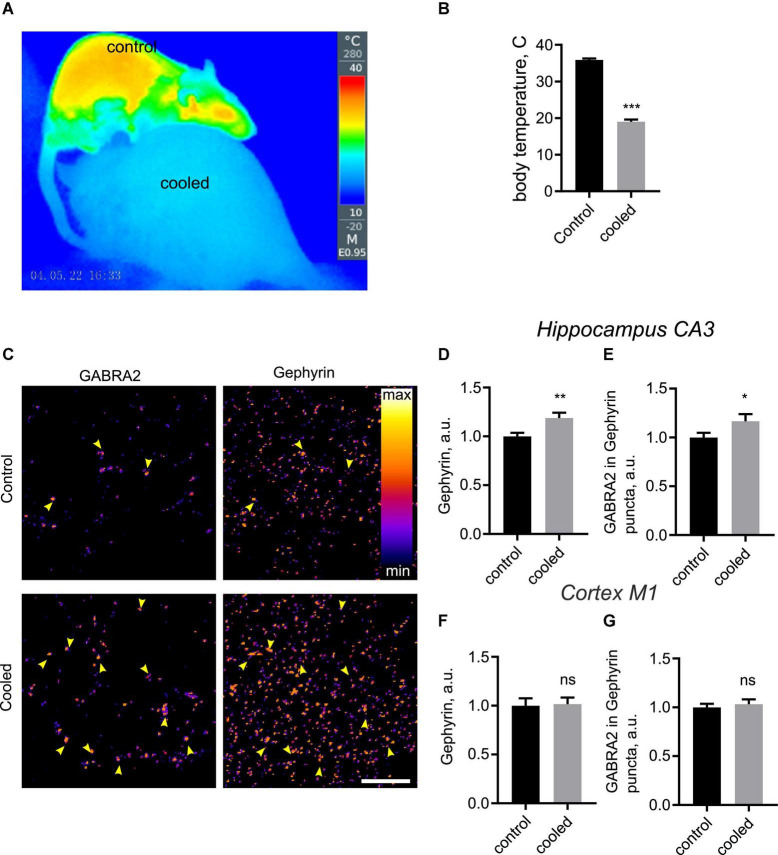
Hypothermia-induced structural remodeling of inhibitory synapses *in vivo*. **(A–C)** Rats were anesthetized, injected with 5′-AMP (cooled group) or water (control group), and kept at 18°C or room temperature for 3 h. Following this, brain sections were immunostained for GABRA2 and Gephyrin. **(A)** A representative thermographic image showing the effect of the hypothermia-inducing treatment on body temperature. Top, control rat; bottom, cooled rat. **(B)** Quantification of body temperature in control and cooled rats. ***P < 0.001, Student *t*-test. **(C)** Representative images of hippocampal CA3 regions from control and cooled groups. Arrowheads denote colocalization between GABRA2 and Gephyrin, indicative of inhibitory synapses. **(D)** Quantification of Gephyrin in Gephyrin-positive puncta in the CA3 region. **P < 0.01, Student *t*-test. **(E)** Quantification of GABRA2 levels in Gephyrin-positive puncta in the CA3 region. *P < 0.05, Student *t*-test (one-tailed). **(F)** Quantification of Gephyrin in Gephyrin-positive puncta in the M1 cortex. ns, not significant, Student *t*-test. **(G)** Quantification of GABRA2 levels in Gephyrin-positive puncta in the M1 cortex. ns, not significant, Student *t*-test. *N* = 30 images, 3 images per animal, 10 animals per group. Scale bar, 10 μm.

To investigate structural plasticity of inhibitory synapses in the physiologically relevant context of intact CNS circuitry, we decided to investigate synaptic remodeling *in vivo*, using 5′-AMP to induce a hypometabolic state (Zhang et al., 2006). Injection of 5′-AMP, but not saline, consistently lowered the core temperature in rats to 18°C (Figures 4A,B), confirming the validity of this protocol for hypothermia induction. We therefore investigated the impact of this treatment on organization of inhibitory synapses in the CA3 region of hippocampus, using immunohistochemistry staining for Geph and GABRA2. Intensity of the Geph-positive puncta was significantly increased in cooled animals relative to the control animals (Figures 4C,D). Furthermore, GABRA2 levels in Geph + puncta were increased in cooled animals relative to the controls as well (Figures 4C,E). These findings confirm that hypothermia does indeed induce postsynaptic enhancement of inhibitory synapses in hippocampus *in vivo*. In contrast to this, hypothermia had no significant effect on intensity of Homer + and Bsn + puncta in the hippocampus, suggesting that excitatory PSD was not affected by hypothermia *in vivo* (Supplementary Figure 3C–E).

Previous evidence has shown loss of structurally defined synapses following hypothermia in the cortex (von der Ohe et al., 2007). We therefore imaged Geph and GABRA2, Homer and Bsn in the primary motor cortex area (M1). Hypotherma did not lead to any significant changes for any of the above proteins (Figures 4F,G and data not shown), suggesting that synaptic structure in the cortex remained stable during hypothermia. Taken together, our findings confirm that inhibitory synapses are specifically enhanced by hypothermia in the hippocampus.

### Proteomic analysis of cortical synaptome

Lack of the effect of hypothermia on cortical synapses suggests that temperature-dependent mechanisms of synaptic plasticity may be restricted to hippocampus. How stable are cortical synapses in hypo- and hypothermia? For a comprehensive investigation of temperature-dependent structural synaptic plasticity in the cortex, we used tandem mass tagging (TMT) proteomics of synapses biochemically purified from dissociated cultures of cortical neurons (Supplementary Figure 4).

The results of proteomic analysis are presented in Supplementary Figure 4 and Supplementary Table 2. Of the 5254 synaptic proteins identified in the hypothermia experiments, 9 and 4 were upregulated and downregulated, respectively (Supplementary Figures 4A,B). Of the 6,382 synaptic proteins identified in the hyperthermia experiments, 11 and 16 were upregulated and downregulated, respectively (Supplementary Figures 4C,D). While some of the significantly affected proteins were associated with neurodegeneration and synaptic function (Supplementary Table 2), none of them were associated with synaptic structure. Taken together, these results confirm broad stability of cortical synaptic structure in response to temperature as evidenced by immunohistochemistry *in vivo*.

## Discussion and conclusion

This study presents a first-to-date systemic investigation of temperature-dependent structural and functional synaptic remodeling in hippocampal neurons. To achieve a comprehensive view of temperature-dependent processes, we considered both cooling (hypothermia) and fever (hyperthermia) separately. While hypothermia and hyperthermia have broadly opposite effects on the presynaptic compartment, the PSD of inhibitory hippocampal synapses is only remodeled by hypothermia, involving a translation-independent mechanism based on actin dynamics.

Firstly, we show that hypothermia elicits diverse effects on multiple key classes of proteins across the synapse, including scaffolding proteins, neurotransmitter transporters and receptors. In contrast to the previously considered simplistic notion of synaptic dismantlement (Popov and Bocharova, 1992; Magariños et al., 2006; von der Ohe et al., 2006, 2007; Peretti et al., 2015), our results reveal a complex pattern of temperature-induced rearrangement of synaptic structure in the hippocampus that may provide a way to optimize or sustain neuronal functionality in response to changing environmental conditions. While our data appears at odds with previous studies in rodents, the discrepancy is likely explained by experimental differences, whereby the majority of previous studies utilized disparate temperature regimes, analytical methodology, and models; for instance, the recent study by Peretti et al. (2015) used mice, focused on the CA1 region, and–importantly–relied on ultrastructural imaging rather than immunostaining, thus not distinguishing between excitatory and inhibitory synapses. The key role of experimental conditions is further highlighted by our observation of excitatory postsynaptic enhancement in cultured cells, but not *in vivo*. Taken together, our data on cooling-induced enhancement on inhibitory synapses reveals a major novel facet of hypothermia response in the brain, with significant implications for understanding the mechanisms of neuroprotection during stroke and TBI.

Another key finding of this work is the profound effect of mild hyperthermia on synaptic structure and function. Considering that fever is the most common indication of a diseased state and cause for medical help (Ogoina, 2011), our data suggests that even short-term episodes of mild (39°C) fever may be associated with significant consequences for neuronal function. Taking into account the key role of synaptic transmission in the developing CNS, these observations assume particularly stark relevance in the context of pediatric fever, which affects the majority of children by the age of 5 years (Barbi et al., 2017). Temperature-induced dysregulation of synaptic plasticity also has implications for association between hyperthermia and adverse neurological outcomes (Lundgren et al., 1994; Thompson et al., 2003; Walter and Carraretto, 2016). Conversely, hyperthermia-associated changes in synaptic BACE2 levels are intriguing in the context of suggested therapeutic benefit of hyperthermia in Alzheimer’s disease (Hunt et al., 2020). These findings pave the way for wider research into synaptic effects of hyperthermia, with particular focus on the mechanisms and the long-term effects of hyperthermia *in vivo* and in relevant disease models.

Our results provide a novel mechanistic insight into the mechanisms of temperature-dependent synaptic plasticity. Remarkably, we find that cooling-induced reorganization of inhibitory PSD can proceed in the absence of protein biosynthesis, suggesting that this form of synaptic plasticity is likely uncoupled from the classical temperature shock response (Lindquist, 1986; Fujita, 1999; Peretti et al., 2015). Instead, our results highlight the importance of temperature-dependent cytoskeletal dynamics (Li and Moore, 2020; Kubota et al., 2021), which is evidenced by the hypothermia-induced actin redistribution. Owing to the paucity of published evidence regarding environmental regulation of actin dynamics *in vivo*, detailed mechanistic understanding of this process will require further study. Although it may be tempting to speculate that actin polymerization in itself may represent an intrinsic temperature sensor, it is likely to be a component of an integrated cell-wide thermometric system alongside temperature-dependent ion channels, gene expression, metabolic rate, and possibly other unknown mechanisms. The armamentarium of cell biological mechanisms involved in environmental response therefore warrants further exploration.

Our work shows significant differences between temperature-dependent synaptic plasticity between brain regions and between experimental settings. Firstly, while inhibitory hippocampal synapse enhancement was observed both in culture and *in vivo*, this was not the case for excitatory synapses, where the small enhancement observed in culture was not matched by the *in vivo* observations. Apart from the variability of immunohistochemistry staining and the small sample size (10 animals/condition), this discrepancy may be related to relative lack of glial cells in the culture vs. the brain, where 68% synapses are associated with glial cells that play a key role in synaptic remodeling *in vivo* (Kikuchi et al., 2020). Secondly, both immunohistochemistry and proteomics have revealed apparent lack of effect of temperature on the synapses in the motor cortex. It is not unlikely that this apparent stability may arise from the diverse identities of cells in the motor cortex; in contrast, both CA3 region and the embryonic hippocampal dissociated cell culture the CA3 region mainly consist of pyramidal cells (Witter, 2007; Wu et al., 2015). It is also possible that the overall abundance of synaptic proteins across the cortex either remains the same or the observed changes are too small to be detected by proteomics. Altogether, these findings reveal different processes of structural synaptic plasticity operating in different brain regions, highlighting the need for further systemic investigation of local synaptic plasticity across the CNS. Based on our data showing temperature-induced remodeling of presynaptic and postsynaptic compartments (Supplementary Figure 5), future investigations will characterize the effects of hypo/hyperthermia on key synaptic structural and functional features *in vivo*, such as cytoskeletal dynamics, membrane trafficking pathways, distinct SV pools, and nanoscale alignment of presynaptic release sites and postsynaptic receptors, leveraging the methodology of electrophysiology and live imaging.

Last but not least, our findings add to an emerging body of evidence regarding the experimental standards for protocols in neurobiology. Although neuroscience experiments were historically carried out at room temperature, deviations from the physiological temperature have since been shown to affect multiple aspects of synaptic structure and function (Micheva and Smith, 2005; de la Peña et al., 2012; Watanabe et al., 2014). In the light of this evidence, the catalog of temperature-dependent changes in synaptic organization presented in this work further underscores the importance of maintaining rigorous experimental conditions for investigation of neuronal function.

## Data availability statement

The original contributions presented in this study are included in the article/Supplementary material, further inquiries can be directed to the corresponding author.

## Ethics statement

This animal study was reviewed and approved by Qingdao University.

## Author contributions

ZF, QW, and OG: study design. ZF, LS, CD, and OG: experiments and analysis. ZF, LS, CD, QW, and OG: writing. All authors contributed to the article and approved the submitted version.
