# Supplementary material for: Temperature-dependent structural plasticity of hippocampal synapses
Source: Front Cell Neurosci. 2022 Oct 19;16:1009970. doi: 10.3389/fncel.2022.1009970 (PMC9627613; doi:10.3389/fncel.2022.1009970)
Supplement: Supplementary file 1 [file Data_Sheet_1.pdf]

Materials and Methods

Materials

Cell culture reagents and AlexaFluor647-conjugated Phalloidin were from Invitrogen (UK). FITC-conjugated CTB, Anisomycin, Jasplakinolide and sundry reagents were from Sigma Aldrich (UK). TTX, NBQX, APV, Gabazine were from Tocris (UK). The list of the antibodies used in this study can be found in **Table 1**.

Table 1. List of the antibodies used in this study

Antigen	Conjugation	Species	Manufacturer	Cat. No.
vGlut1		rabbit	Synaptic Systems	135302
Homer		rabbit	Synaptic Systems	160002
Bassoon		mouse	Abcam	82958
Cav2.1		rabbit	Synaptic Systems	152103
Piccolo		rabbit	Synaptic Systems	142002
Gephyrin		mouse	Synaptic Systems	147011
GABRA2		Rabbit	Synaptic Systems	224103
Syt-1		mouse	Synaptic Systems	105311
Rabbit IgG	Cy5	donkey	Jackson Immuno	711-175-152
Mouse IgG	AlexaFluor488	goat	Invitrogen	A-11001
Mouse IgG	AlexaFluor633	goat	Invitrogen	A-21052
Rabbit IgG	AlexaFluor488	goat	Bioss	bs-0295G-AF488
Mouse IgG	AlexaFluor594	goat	SolarBio	K1031G-AF594

Neuronal culture

Dissociated hippocampal and cortical neuronal cultures were prepared from E18 rat embryos and grown according to the Brewer protocol in Neurobasal medium with added GlutaMax and B-27 supplement. All experiments involving neurons were carried out at 16-21 days in vitro. Cells were plated onto 13 mm round glass coverslips (thickness 1.5) placed in 35 mm Petri dishes (4/dish). To reduce variability, each experiment was carried out using the coverslips cultured within the same Petri dish. All experimental protocols were performed in accordance with the guidelines of the relevant Research Ethics Committee.

Hypothermia and hyperthermia treatment in vitro

Two empty 200μm pipette tip rack holders were filled with 20ml distilled water each; and an appropriate area (circa 5sq.cm/coverslip) of the surface was covered with Parafilm on each rack holder. Coverslips with live dissociated neurons or slices were placed upside up onto the Parafilm-covered surfaces and 100ul of conditioned culture medium with 50mM HEPES was added to each coverslip. The lids of the rack holders were closed. One rack holder remained at the laboratory bench for the duration of the experiment, whereas the other was placed in the standard incubator for bacterial culture (i.e. without CO_2) set to 37°C. A

thermometer was placed at each location, and temperature was periodically monitored. RT was consistently measured between 19 and 22°C. Following the required period of incubation, both holders were kept at the bench for 10-20 min prior to fixation with lids open to equilibrate their temperature.

For the heating procedure, one cell culture incubator was set to 37 and the other at 39°C, and the coverslips were placed on Parafilm and incubated in the conditioned culture medium for the required length of time.

Hypothermia treatment in vivo

Ten male Sprague Dawley rats (body weight 230-250 g) were randomly divided into the “cooled” group and the “control” group. 5’-AMP was freshly dissolved in a phosphate buffer and adjusted to pH7.4. The rats in the “cooled” group were injected intraperitoneally with the equivalent volume of the 5’-AMP solution to the final concentration of 1 g per kg body weight and then maintained at 16-18 ℃ for 3 hours. The rats in the “control” group were injected intraperitoneally with the equivalent volume of phosphate buffer (5 ml per kg body weight) and then maintained at 16-18 ℃ for 3 hours. Rat body temperature was measured by a thermal imager at the start and the end of the experiment. The experiment was repeated one more time as described above.

Immunocytochemistry staining

After treatment, coverslips were fixed with 4% PFA in PBS for 15−20min at room temperature (RT) and permeabilized in 0.3% Triton-X100 in PBS supplemented with 5% horse serum for 10 min. Subsequent incubations were carried out in the permeabilization buffer. Coverslips were incubated with appropriate primary antibodies for 60 min at RT, washed 4 times in PBS and incubated with AlexaFluor-488 and AlexaFluor-647-conjugated secondary antibodies as appropriate at a concentration of 0.3 µg/mL each for 60 min at RT. Coverslips were then mounted in Fluoromount-G mounting medium (Southern) and stored at 5C until imaging.

For live surface labelling of SV cycling, after treatment, cells were incubated with 1/100 anti-Syt-1 antibody in presence of either 0.2 uM TTX or 50 mM KCl in conditioned culture at RT for 15min. Following labelling, coverslips were either fixed at RT to or returned to 37C for 15min, then fixed at RT.

Immunohistochemistry staining

After treatment, rats were anaesthetised with isoflurane and then perfused transcardially with ice-cold PBS (pH7.4). The brains were dissected out and kept in -80°C. Sagittal sections (50 um) were cut at -20℃ with a cryostat (Leica CM1950) then fixed with 4% PFA in PBS for 15 mins at RT and washed 10 mins for 3 times in PBS. Subsequent sections were collected in PBS and stored at 4°C for future use. Sections were incubated in blocking buffer (5% goat serum, 0.3% Triton-X 100 in PBS) for 1h at RT, and incubated for 36-48h at 4℃ with primary antibodies diluted in blocking buffer. Sections were washed 10 mins for 3 times in PBS, followed by incubating with AlexaFlour-488 and AlexFlour-594-conjugated secondary antibodies for 70 mins at RT, and washed 10 mins for 3 times with PBS. Then all sections

were mounted onto positively-charged microscope slides and covered in DAPI-containing mount media (Solarbio).

Confocal microscopy imaging

Samples were imaged on a Zeiss LSM710 and Nikon Eclipse Ti2 laser confocal microscopes equipped with a standard set of lasers and objectives. The imaging systems were controlled by ZEN and NIS Elements 2.0 software respectively.

The imaging parameters for Zeiss LSM710 were as follows. Regions of interest sized 1024x1024 pixels (65.8 nm/pixel) were imaged at speed 7 with the averaging setting 2. Pinhole size was kept to 1-2 Airy units. Excitation laser wavelengths were 488 and 633nm. Bandpass filters were set at 500−550 (AlexaFluor488) and 650−750nm (AlexaFluor647). Image acquisition was carried out at the 12-bit rate. Settings were optimized to ensure appropriate dynamic range, low background and sufficient signal/noise ratio. For CTB binding assay, cells were incubated with 1/500 AlexaFluor555-conjugated CTB in culture medium for 10 min at RT, then fixed and processed for immunostaining and confocal microscopy as detailed above.

The imaging parameters for Nikon Eclipse Ti2 were as follows. Serial confocal z stack images (1.25 um step for 5 um at 512 x512 pixels, zoom 1 or 0.5 um step for 2 um at 512 x512 pixels, zoom 2) were acquired with a 100x/1.45 Oil objective (Plan APO λ). Pinhole size was kept to 1.0 Airy units. Excitation laser wavelengths were 488 and 561nm. Bandpass filters were set at 500−550 (AlexaFluor488) and 570−620nm (AlexaFluor594). Settings were optimised to ensure appropriate dynamic range, low background and sufficient signal/noise ratio.

Image analysis

To identify individual synapses, images were binarized in ImageJ using the “Moments” setting, and particles were counted automatically using the “Analyze Particles” command across the whole image. Binarized data from Bsn, Homer and Gephyrin immunostaining signals was used for determination of total, excitatory and inhibitory synapses respectively. Signal intensities were quantified for each synaptic punctum using the Region Of Interest (ROI) Manager function of ImageJ. To avoid rare overlap of multiple synapses, only ROIs with areas ranging from 0.1 to 2μm^2 were included in further analysis. All values of circularity were included in analysis. Individual ROIs were then combined into one compound ROI using the “Combine” and “Add” functions of the ROI Manager interface, whereupon quantification of mean signal intensity in each channel was performed using the “Measure” function. Since background fluorescence intensity was typically less than 1% of the median fluorescence in each channel within a ROI, background subtraction did not significantly affect the measurements and was not performed.

Proteomic analysis

Following the in vitro hypo and hyperthermia treatments, cortex neuronal cultures were processed by Synaptic Protein Extraction Reagent (SynPER, Fisher Scientific) to isolate the synaptosome fraction. Tandem Mass Tagging (TMT) proteomic analysis of the synaptosomal

fraction was performed and analysed by Luming Biotechnology (Shanghai, China) as briefly described below.

For labelling, the lyophilised samples were resuspended in 100 μL 200 mM TEAB and 40 μL of each sample were transferred into new tubes for labelling. 88μl of acetonitrile were added to labelling reagent vial at room temperature. The centrifuged reagents were dissolved for 5 min and mixed for centrifugation; this step was repeated once more. 41 μL of the labelling reagent solution was added to each sample for mixing. The tubes were incubated at room temperature for 1 h. To terminate the labelling reaction, 8 μL of 5% hydroxylamine were added to each sample and incubated for 15 min. The labelled sample solutions were lyophilized and stored at -80 ℃.

Reversed-phase separation was performed on an 1100 HPLC System (Agilent) using an Agilent Zorbax Extend RP column (5 μm, 150 mm × 2.1 mm). Mobile phases A (2% acetonitrile in HPLC water) and B (98% acetonitrile in HPLC water) were used for RP gradient. The solvent gradient was set as follows: 0~8 min, 98% A; 8~8.01 min, 98%~95% A; 8.01~48 min, 95%~75% A; 48~60 min, 75~60% A; 60~60.01 min, 60~10% A;60.01~70 min, 10% A; 70~70.01 min, 10~98% A; 70.01~75 min, 98% A. Tryptic peptides were separated at an fluent flow rate of 300μL/min and monitored at 210 and 280 nm. Samples were collected for 8-60 minutes, and eluent was collected in centrifugal tube 1-15 every minute in turn. Samples were recycled in this order until the end of gradient. The separated peptides were lyophilized.

All analyses were performed by a Q-Exactive mass spectrometer (Thermo, USA) equipped with a Nanospray Flex source (Thermo, USA). Samples were loaded and separated by a C18 column (15 cm × 75 μm) on an EASY-nLC TM 1200 system (Thermo, USA). The flow rate was 300 nL/min and linear gradient was 60 min (0~38 min, 8-22% B; 38~50 min, 22-36% B; 50~54 min, 36-85% B; 54~60 min, 85%B; mobile phase A = 0.1% FA in water and B = 90% ACN/0.1% FA in water). Full MS scans were acquired in the mass range of 300 – 1600 m/z with a mass resolution of 70000 and the AGC target value was set at 1e6. The ten most intense peaks in MS were fragmented with higher-energy collisional dissociation (HCD) with NCE of 32. MS/MS spectra were obtained with a resolution of 17500 with an AGC target of 2e5 and a max injection time of 80 ms. The Q-E dynamic exclusion was set for 30.0 s and ran under positive mode.

ProteomeDiscoverer (v.2.4) was used to search all of the Q Exactive raw data thoroughly against the sample protein database. Database search was performed with Trypsin digestion specificity. Alkylation on cysteine was considered as fixed modifications in the database searching.

Statistical analysis

All the experiments were performed in at least 3 independent biological replicates, with five fields of view per condition selected for analysis. All of the synapses automatically detected within these fields of view were included in the analysis. Statistical analysis was carried out using the Prism 6.0c software package (GraphPad Software). Data distributions were assessed for normality using d’Agostino and Pearson omnibus normality test. For normally distributed datasets, Student’s t-test, 1-way ANOVA and Dunnett’s post-test were used to assess statistical significance as appropriate; for not normally distributed datasets,

Mann-Whitney rank test was used for assessing statistical significance. Datasets were presented as scatter dot plots with line at median and cumulative probability plots as appropriate.

Table 4. Selected synaptic proteins significantly changed by hypo/hyperthermia

Protein	Protein name	Significance	Reference
D3ZVT2	THADA	Associated with cold adaptation; +selection in human vs neanderthal	(Green et al., 2010; Moraru et al., 2017)
Q6IE75	beta -secretase 2	Processing of beta-amyloid; Alzheimer's disease	(Abdul-Hay et al., 2012; Wang et al.)
Q9WTQ1	Protein Kinase D1	Regulates synaptic function	(Cen et al., 2018)
A0A0G2JWX9	Clca1	Activated by exptrasynaptic NMDARs; regulates pro-death pathways	(Zhang et al., 2007)
Q5FVN2	TMEM41B	essential factor for SARS-CoV-2 replication	(Trimarco et al., 2021)
P55926	Acid sensing ion channel 1	Regulates synaptic plasticity	(Yermolaieva et al., 2004)
Q9WUQ3	Oligodendrocyte transcription factor 1		
P09626	H^+/K^+ ATPase	Blockers are associated with dementia	(Ortiz-Guerrero et al., 2018)
Q66H98	Cavin 2	Clathrin-independent endocytosis	(Chaudhary et al., 2014)

P42930	Heat shock protein 27	Protective effects in neurodegeneration	(Lyon and Milligan, 2019)
D3ZNJ9	Ubiquitin ligase TRIM7	Regulates signalling	(Chakraborty et al., 2015)
A0A0G2K9G6	Stromalin	Controls SV numbers, restricts memory	(Phan et al., 2019)
Q6AY56	Alpha tubulin 8	Binds phosphorylated Tau	(Drummond et al., 2020)

Supplementary Figures

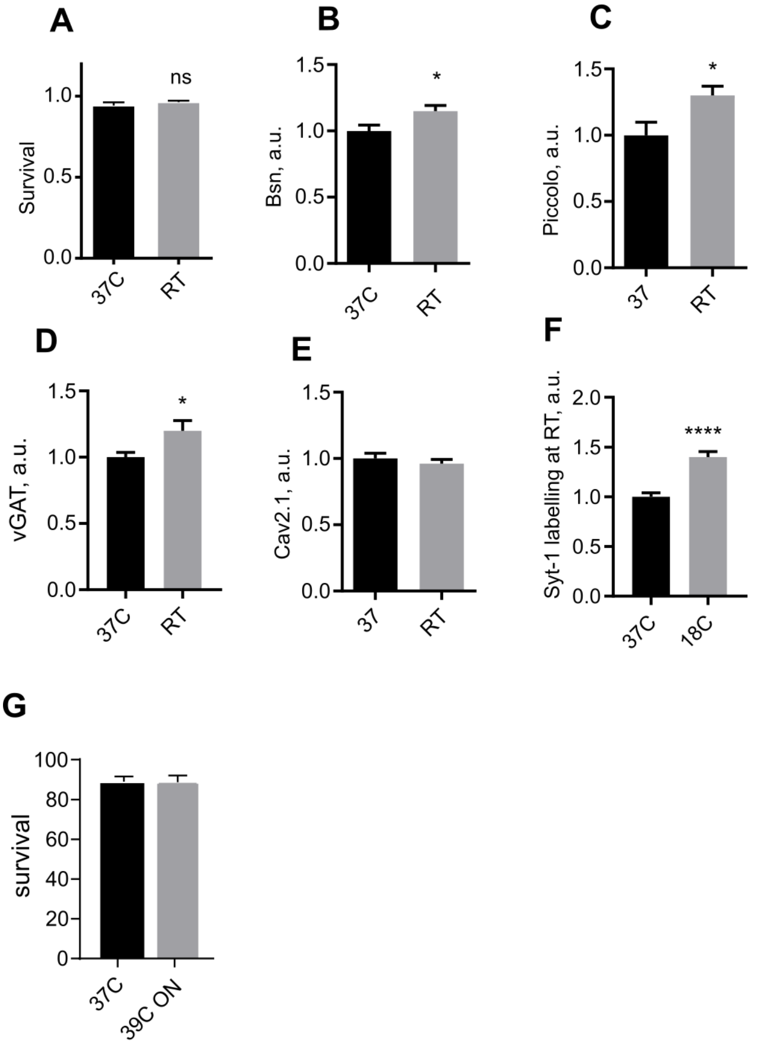

Figure S1. Additional data related to Figures 1&2. A, Hypothermia does not affect neuronal survival. Cells were incubated at 37°C or RT for 3h and survival was quantified using Trypan Blue exclusion assay. N=15 fields of view, 3 independent experiments. ns>0.05, Student t-test. **B**, Effect of hypothermia on synaptic enrichment of Bassoon. N=25 fields of view from 5 independent experiments. **C**, Effect of hypothermia on synaptic enrichment of Piccolo. N=10 fields of view from 2 independent experiments. **D**, Effect of hypothermia on synaptic levels of vGAT. N=25 fields of view from 5 independent experiments. **E**, Effect of cooling on synaptic enrichment of Cav2.1. N=15 fields of view from 3 independent experiments. **F**, Labelling with anti-Syt-1 antibody at RT is increased following hypothermia. Cells were incubated at 37°C or RT for 3h and survival was quantified using Trypan Blue exclusion assay. N=15 fields of view, 3 independent experiments. *P<0.05, ****P<0.01, Student t-test. **G**, Hyperthermia does not affect neuronal survival. Cells were incubated at 37°C or 39C overnight and survival was quantified using Trypan Blue exclusion assay. N=15 fields of view, 3 independent experiments. ns>0.05, Student t-test.

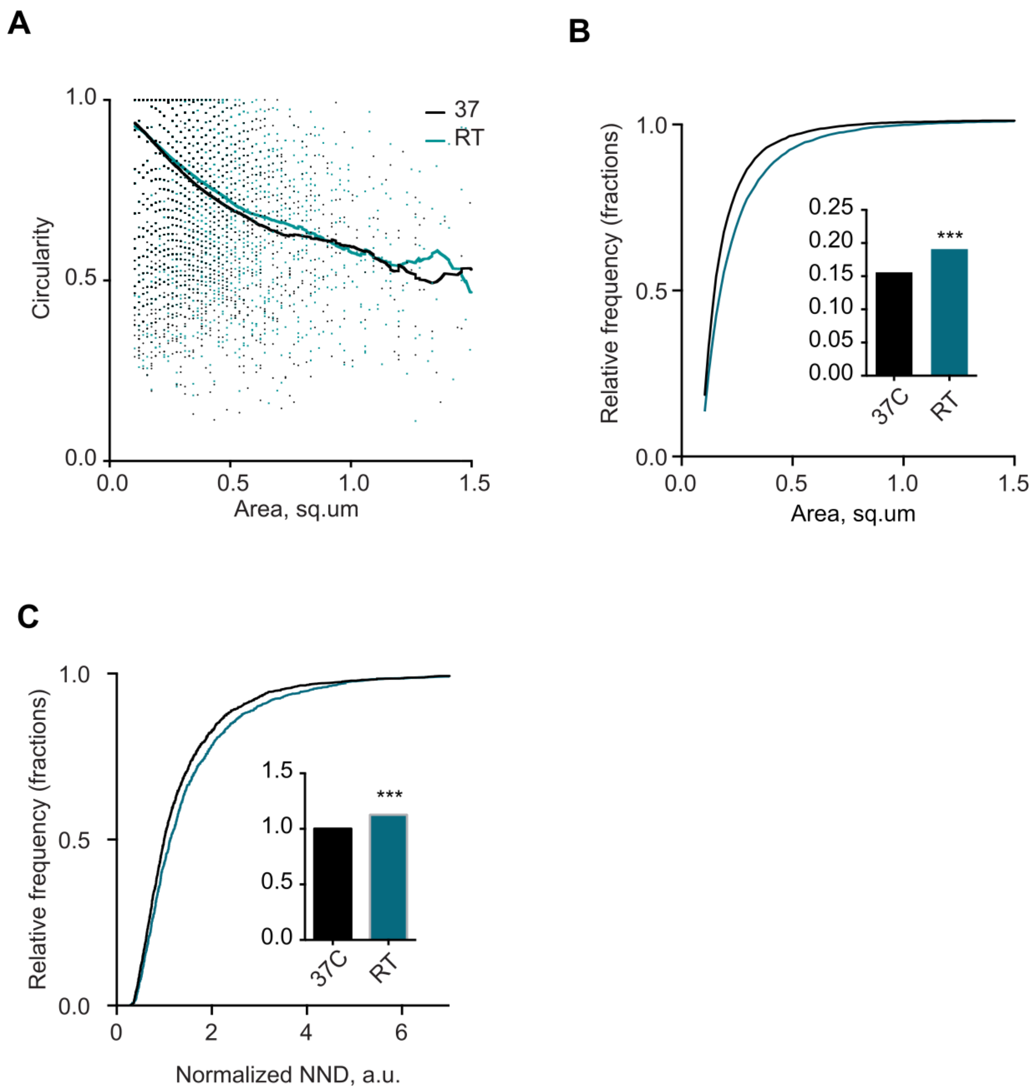

Figure S2. Hypothermia-induced changes in the geometry and distribution of the inhibitory PSD (Gephyrin-positive puncta). A, The plot of circularity against area, showing LOWESS fits. N=9652 synapses (37°C) and 7156 synapses (RT), 3 independent experiments. **B**, Cumulative probability plot for area. Inset, median values. **C**, Cumulative probability plot for nearest neighbour distances, normalized to median values in the 37°C control sample. Inset, median values. N= 2096 synapses (37°C) and 1912 synapses (RT), 3 independent experiments. ***P<0.001, Mann-Whitney test.

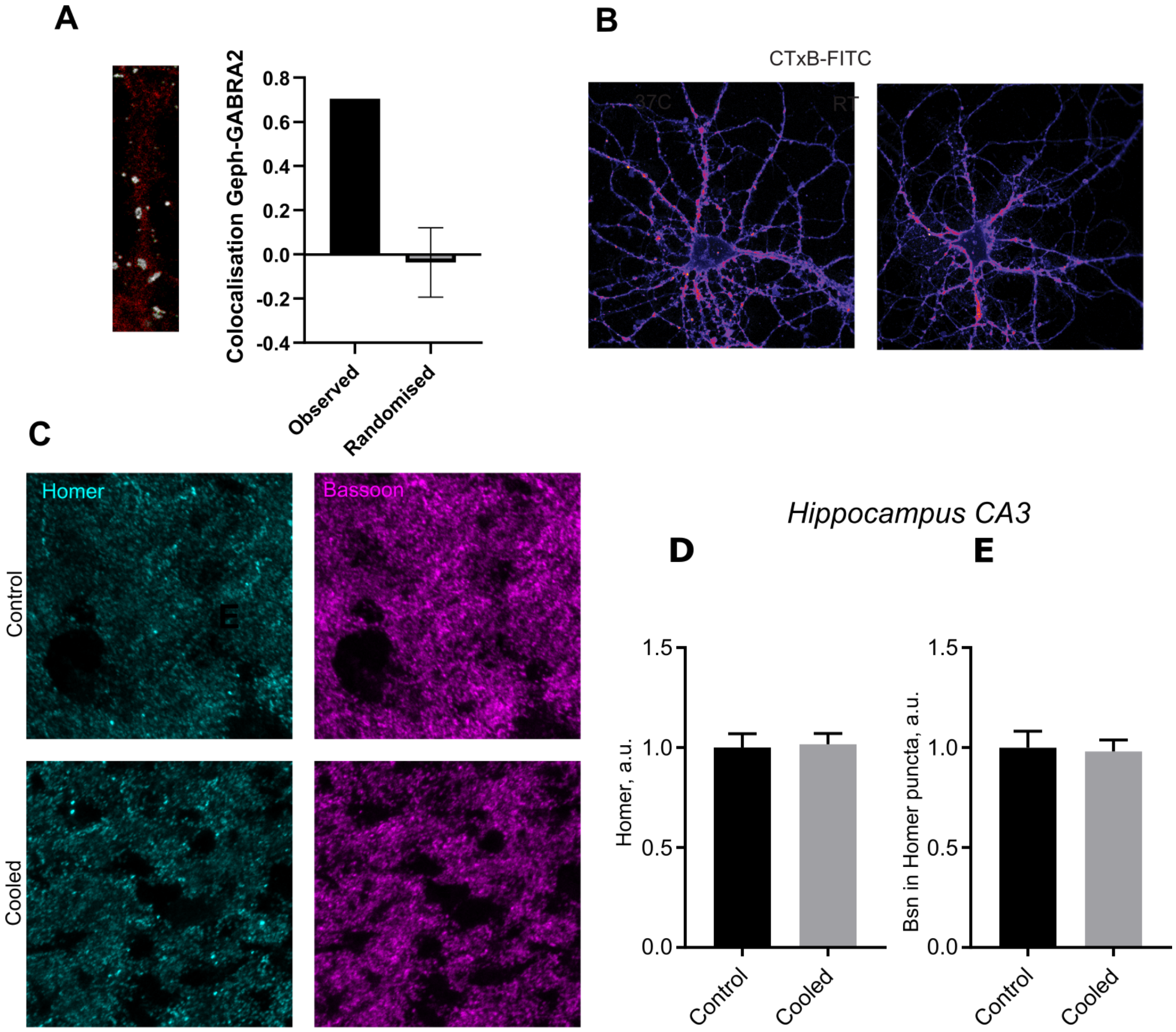

Figure S3. Additional data related to Figures 3&4. A, Image from Figure 3D (right panel) was analysed by the Colocalization Test and compared to 25 interactions of randomisation. **B**, Cells were subjected to RT for 3h, then stained with FITC-labelled Cholera toxin B subunit to visualise plasma membrane. **C**, Rats were anaesthetised, injected with 5'-AMP (cooled group) or water (control group), and kept at 18°C or room temperature for 3 hours. Following this, brain sections were immunostained for Homer and Bsn. **D**, Quantification of Homer positive puncta. **E**, Quantification of Bsn levels in Homer-positive puncta. N=30 images, 3 images per animal, 10 animals per group.

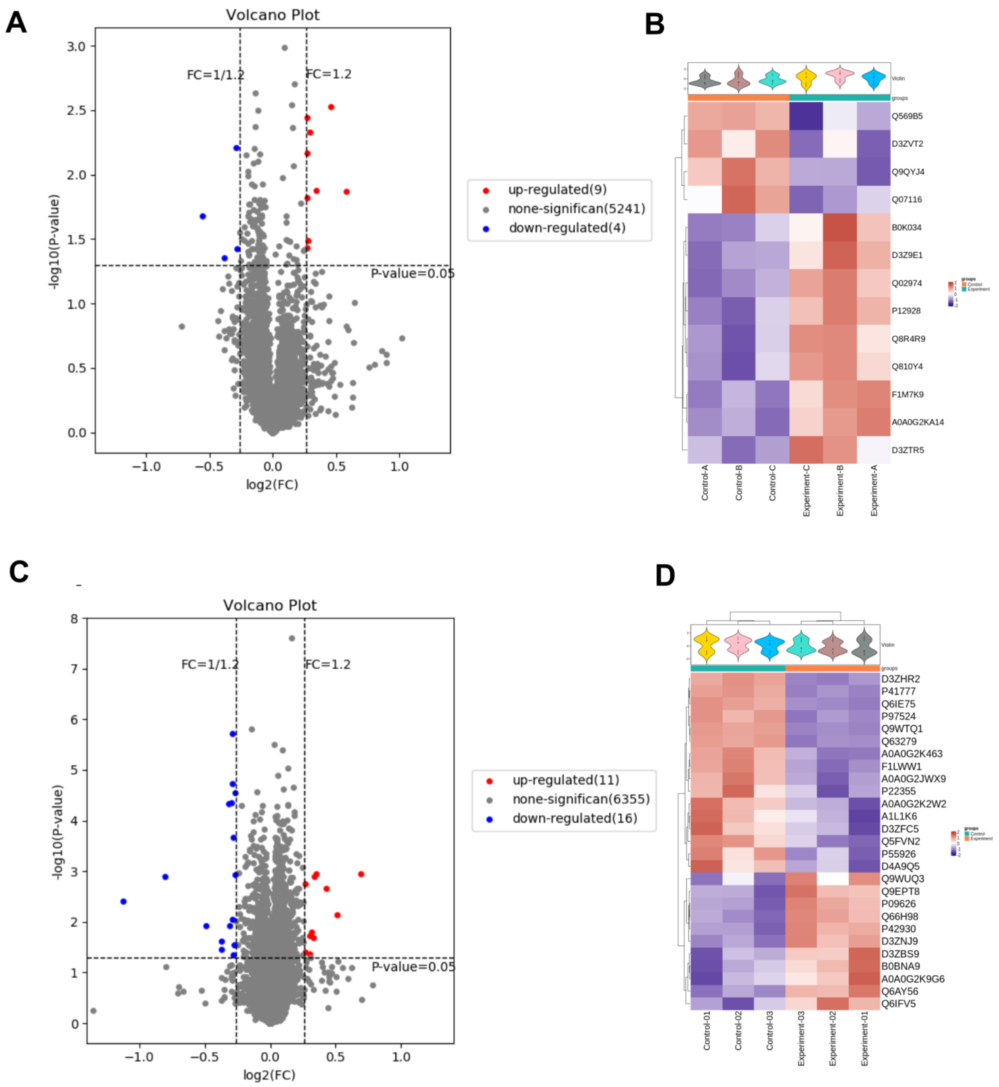

Figure S4. Proteomic analysis of synaptosomes following hypo- and hyperthermic treatment. A-B, Cells were incubated at 37°C or RT for 3h, and synaptosomal fractions were analysed by Tandem Mass Tagging proteomics. **A**, Volcano plot of all proteins. **B**, Heat map showing 13 differentially expressed proteins. **C-D**, Cells were kept at 37°C or 39°C overnight, and synaptosomal fractions were analysed by Tandem Mass Tagging proteomics. **C**, Volcano plot of all proteins. **D**, Heat map showing 27 differentially expressed proteins.

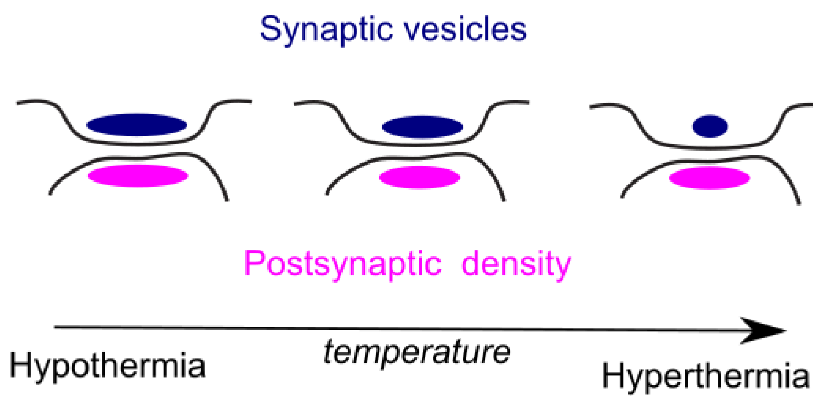

Figure S5. Proposed simplified model of temperature-dependent synaptic remodelling in dissociated hippocampal cell cultures. *Left*, hypothermia results in enhancement in both SV and PSD, thereby preserving the matching between synaptic function. *Right*, mild hyperthermia diminishes SV without affecting PSD, altering the balance between the presynaptic and postsynaptic function.
